# Inhibition of human cytomegalovirus immediate early gene expression and growth by a novel RNase P ribozyme variant

**DOI:** 10.1371/journal.pone.0186791

**Published:** 2017-10-23

**Authors:** Xu Sun, Weijie Chen, Lingling He, Jingxue Sheng, Yujun Liu, Gia-Phong Vu, Zhu Yang, Wei Li, Phong Trang, Yu Wang, Rong Hai, Hua Zhu, Sangwei Lu, Fenyong Liu

**Affiliations:** 1 College of Life Science and Technology, Jinan University, Guangzhou, Guangdong, China; 2 School of Public Health, University of California, Berkeley, CA, United States of America; 3 School of Medicine, St. George’s University, Grenada, West Indies; 4 School of Pharmacy, Shandong University of Traditional Chinese Medicine, Jinan, Shandong, China; 5 Guangzhou Qinheli Biotechnologies, Inc., Guangzhou, Guangdong, China; 6 Jiangsu Affynigen Biotechnologies, Inc., Taizhou, Jiangsu, China; 7 Taizhou Institute of Virology, Taizhou, Jiangsu, China; University of St Andrews, UNITED KINGDOM

## Abstract

We have previously engineered new RNase P-based ribozyme variants with improved in vitro catalytic activity. In this study, we employed a novel engineered variant to target a shared mRNA region of human cytomegalovirus (HCMV) immediate early proteins 1 (IE1) and 2 (IE2), which are essential for the expression of viral early and late genes as well as viral growth. Ribozyme F-R228-IE represents a novel variant that possesses three unique base substitution point mutations at the catalytic domain of RNase P catalytic RNA. Compared to F-M1-IE that is the ribozyme derived from the wild type RNase P catalytic RNA sequence, the functional variant F-R228-IE cleaved the target mRNA sequence in vitro at least 100 times more efficiently. In cultured cells, expression of F-R228-IE resulted in IE1/IE2 expression reduction by 98–99% and in HCMV production reduction by 50,000 folds. In contrast, expression of F-M1-IE resulted in IE1/IE2 expression reduction by less than 80% and in viral production reduction by 200 folds. Studies of the ribozyme-mediated antiviral effects in cultured cells suggest that overall viral early and late gene expression and viral growth were inhibited due to the ribozyme-mediated reduction of HCMV IE1 and IE2 expression. Our results provide direct evidence that engineered RNase P ribozymes, such as F-R228-IE, can serve as a novel class of inhibitors for the treatment and prevention of HCMV infection. Moreover, these results suggest that F-R228-IE, with novel and unique mutations at the catalytic domain to enhance ribozyme activity, can be a candidate for the construction of effective agents for anti-HCMV therapy.

## Introduction

Human cytomegalovirus (HCMV) is a member of herpesviridae family [1]. Seven other human viruses in the family are: herpes simplex virus 1 and 2 (HSV-1 and 2), Varicella Zoster virus (VZV), Epstein-Barr virus (EBV), human herpesvirus 6 and 7, and Kaposi sarcoma-associated herpesvirus (KSHV) [2]. HCMV causes numerous diseases in humans especially in immunocompromised individuals, including AIDS patients [1]. Thus, creating novel and useful antiviral treatments is essential for combating HCMV infection.

The scientific communities have recognized ribozymes as promising antiviral agents to "silence” genes by creating a cleavage to the viral mRNA sequences and inhibiting viral growth [3–5]. Also, ribozymes are believed to work better than conventional antisense approaches by acting as catalysts and producing irremediable damage of the targeted RNAs. Therefore, we researched anti-HCMV effects of ribonuclease P-based ribozymes in vitro and in vivo in this study.

**Fig 1:**
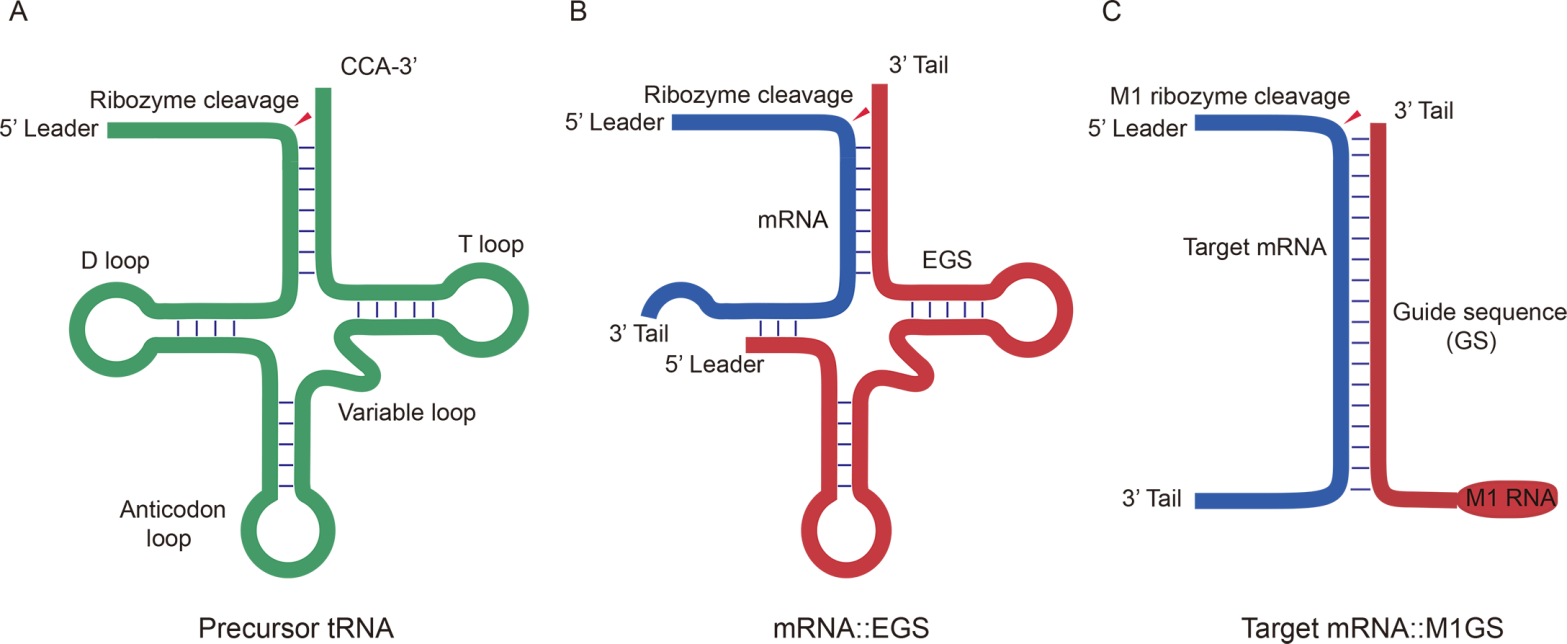
Substrates for M1 RNA and RNase P. (A) pre-tRNA; (B) A target mRNA bound to an EGS; (3) a target mRNA bound to an M1GS ribozyme.

During the tRNA maturation process, ribonuclease P (RNase P) hydrolyzes and removes a 5’ leader sequence from a tRNA precursor (pre-tRNA) (Fig 1) [6,7]. In *Escherichia coli*, RNase P, a ribonucleoprotein complex, consists of a C5 subunit (a protein subunit) and an M1 RNA (a catalytic RNA subunit) [8]. Extensive studies have been carried out to understand the catalytic mechanism and substrate binding of RNase P ribozymes [6,7]. The three-dimensional structures of several RNase P catalytic RNAs were investigated [9]. RNase P catalytic RNAs can be folded into a catalytic domain (C domain), which contains several conserved regions such as nucleotides 1–61 and 270–332; and a specificity domain (S domain) participating in binding of tRNA substrate, which includes several regions such as nucleotides 73–100 and 110–200 [10,11].

Altman and colleagues have demonstrated that M1 ribozyme can hydrolyze an mRNA target provided that an external guide sequence (EGS) is designed to form a hybrid with the mRNA resulting in a specific complex comprising of the 5’ leader sequence, the 3’ CCA sequence, the acceptor stem and the T-stem of a tRNA [12,13] (Fig 1). Thus, we can engineer a ribozyme (called M1GS RNA), which is capable of interacting selectively with an mRNA target of interest, by constructing a covalent linkage between the 3’ end of M1 RNA and a guide sequence (GS) [14,15] (Fig 1). M1GS ribozymes have been demonstrated to cleave human and viral mRNAs and suppress expression of viral genes and growth of various human viruses including HSV-1, HCMV, hepatitis B virus (HBV), and human immunodeficiency virus (HIV-1) [16–19].

To optimize the usage of M1GS ribozymes as an effective gene-targeting tool, it is necessary to enhance M1GS RNA efficacy *in vitro* and *in vivo*. Taking advantage of an *in vitro* selection procedure [20–22], we have isolated new M1GS ribozyme variants with more catalytic efficiency than wild-type M1 RNA [23]. In this report, we engineered an novel M1GS variant, F-R228-IE, to target the shared region of HCMV IE1 and IE2 mRNAs, which are essential for viral infection [1], and studied its catalyzing activity *in vitro* and anti-HCMV effect in cultured cells. Our results suggest that F-R228-IE is at the minimum 100 times more effective than the M1GS ribozyme originating from the wild type M1 RNA in slicing the target HCMV mRNA sequence *in vitro*. Compared to that of M1 RNA, the catalytic domain of F-R228-IE contains three unique point mutations: G_59_ -> A_59_, C_123_ -> U_123_, and C_326_ -> U_326_ [23]. How these mutations enhance the in vitro cleavage activity of the ribozyme is not known. Our results also showed that the engineered ribozyme is more efficient in stopping IE1 and IE2 expression and replication of HCMV in cultured cells. Under the expression of the ribozyme variant, we observed that expression of IE1 and IE2 was reduced to 1–2%, and HCMV replication was reduced by 50,000 fold. All of these results provide direct evidence that the isolated ribozyme variant is effective in reducing the gene expression and production of HCMV. Furthermore, our study shows that the new engineered ribozyme variant can be utilized as a candidate for the construction of effective agents for anti-HCMV treatment.

## Results

### Efficient *in vitro* cleavage of HCMV mRNA by engineered RNase P ribozymes

IE1 and IE2 are the major transcriptional factors which regulate viral early (β) and late (γ) genes expression [1]. These two proteins share the same 85 amino acids resulting from differential splicing and polyadenylation of mRNAs which are initiated by a common promoter [24]. In our study, ribozymes were designed to target the shared region of the IE1 and IE2 mRNAs to inhibit the expression of these two proteins. Using a cultured cell-based mapping approach [14,25,26], we identified the shared regions (IE1/2 mRNA) of the IE1 and IE2 mRNAs subjected to modification by dimethyl sulphate (DMS). Based on these results, we designated a position 43 nucleotides downstream from the translation initiation codon of the IE1 and IE2 mRNAs as the cutting site for MG1S ribozyme. This position, which was among the regions that are the most open for DMS modification in HCMV-infected cells (data not shown), is believed to be potentially accessible and available to be bound by a ribozyme.

After performing a selection *in vitro*, we had previously identified M1GS RNA variants with more efficiency in slicing the thymidine kinase (TK) mRNA of HSV-1 than the ribozyme originated from the wild type M1 RNA [23]. Mutations were introduced into the catalytic and substrate binding domains of M1 RNA, which include regions such as nucleotides 30–61, 110–140, and 290–332 [10,11], and those ribozymes that exhibited efficient cleavage activities were isolated [23]. R228 and the functional ribozyme originated from this variant, F-R228-IE, were among the most efficient M1GS RNAs in slicing the TK mRNA in addition to the IE1/2 mRNAs *in vitro* (see below, Table 1). Compared to M1 RNA, three point mutations were found in R228: G_59_ -> A_59_, C_123_ -> U_123_, and C_326_ -> U_326_ [23]. In this study, we investigated the *in vitro* catalytic activity of F-R228-IE and examined its ability as an inhibitor of IE1 and IE2 expression in cells infected with HCMV.

**Table 1:** Overall cleavage rate [(k_cat_/K_m_)^s^] and binding affinity (K_d_) in reactions of substrates ie-39 with RNase P ribozymes. The values are derived from experiments that were performed in triplicate and repeated three times. “ND”: not determined.

Enzyme	k_cat_/K_m_ (μM^-1^·min^-1^)	K_d_ (nM)
F-M1-IE	0.18±0.06	0.35±0.05
C-M1-IE	<5x10^-6^	0.38±0.06
F-R228-IE	18.5.±0.06	0.32±0.06
C-R228-IE	<5x10^-6^	0.31±0.05
F-M1-TK	<5x10^-6^	ND

We engineered functional F-R228-IE and F-M1-IE by covalently joining the 3’ end of R228 RNA and M1 RNA to an 18nt long guide sequence complementary to the designated mRNA sequence, respectively. Similarly, control ribozymes C-M1-IE and C-R228-IE were engineered from an inactive ribozyme mutant C102. C102 RNA originated from M1 RNA, and its P4 catalytic domain contains various point mutations and is at least 1x10^5^ fold less active compared to M1 RNA [27]. C-M1-IE and C-R228-IE were anticipated to be not active due to several nucleotide alterations in the domain responsible for catalytic activity [27,28]. These four ribozymes all shared the identical guide sequence complementary to the targeted IE1/2 mRNAs.

In the presence of F-R228-IE and F-M1-IE, we observed the cleavage of RNA substrate ie-39, which consists of 39 nucleotides from the targeted HCMV mRNA (Table 1). To the contrary, since there are point mutations in the catalytic center region of C-M1-IE and C-R228-IE, we barely detected cleaving of the substrate mRNA sequence in the presence of these two ribozymes (Table 1). The results of the overall cleavage rate (k_cat_/K_m_)^s^ suggest that F-R228-IE has 100 times higher catalytic activity than F-M1-IE, which originated from wild-type M1 RNA (Table 1). The cleavage activity of ribozyme F-R228-IE was also about 100-fold higher than that of F-M1-IE using a 500-nucleotide long IE1 mRNA substrate (data not shown). These results suggest that F-R228-IE is more efficient in cleaving the full-length IE1 and IE2 mRNAs compared to F-M1-IE.

To study whether the difference in binding affinities resulted in different cleavage efficiencies of these ribozymes, gel shift assays were used to extrapolate the binding affinities of ribozymes to substrate ie-39, measured as the dissociation constant (K_d_). The binding affinities of C-R228-IE and C-M1-IE were comparable to those of F-R228-IE and F-M1-IE (Table 1). Thus, we used C-R228-IE and C-M1-IE as antisense effect controls because these two ribozymes displayed similar affinity to ie-39 as F-R228-IE and F-M1-IE but are inactive.

### Expression of ribozyme in human cells

**Fig 2:**
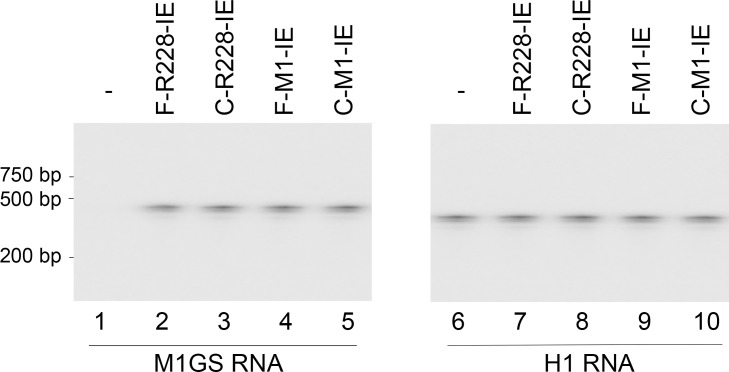
Ribozyme expression in cells assayed using Northern blot analysis. RNA fractions (40 μg) from the parental U251 cells (-) and cells expressing different ribozymes (F-M1-IE, C-M1-IE, F-R228-IE, and C-R228-IE) were hybridized to probes for detection of human H1 RNA (internal control) (lanes 6–10) and ribozymes (lanes 1–5).

We used the LXSN retroviral vector expression system to express functional ribozymes F-R228-IE and F-M1-IE as well as controlled ribozymes C-R228 and C-M1-IE in human cells [14,27–29]. We transfected PA317 cells [29] with LXSN-M1GS DNAs to generate retroviruses that contained the M1GS RNA encoding sequences and to create the cell lines that expressed M1GS ribozymes [29]. These retroviruses were then used to infect human U251 cells, which are permissive for HCMV lytic infection and replication. The cells that expressed the ribozymes and retroviruses were cloned. For three months, there was little difference in cell viability and growth between the parental U251 cells and the cell lines that contained LSXN vector DNA (data not shown), implying no significant cytotoxicity from ribozyme expression. A DNA probe that is complementary to M1 RNA was used in Northern blot experiments to reveal the expression of ribozymes in different cell clones (Fig 2, lanes 1–5). Human H1 RNA expression served as the internal control (Fig 2, lanes 6–10). In subsequent experiments, we studied the cell lines that had similar ribozyme expression levels in cultured cells.

### More effective inhibition of HCMV IE1 and IE2 expression in cells expressing the ribozyme variant

**Fig 3:**
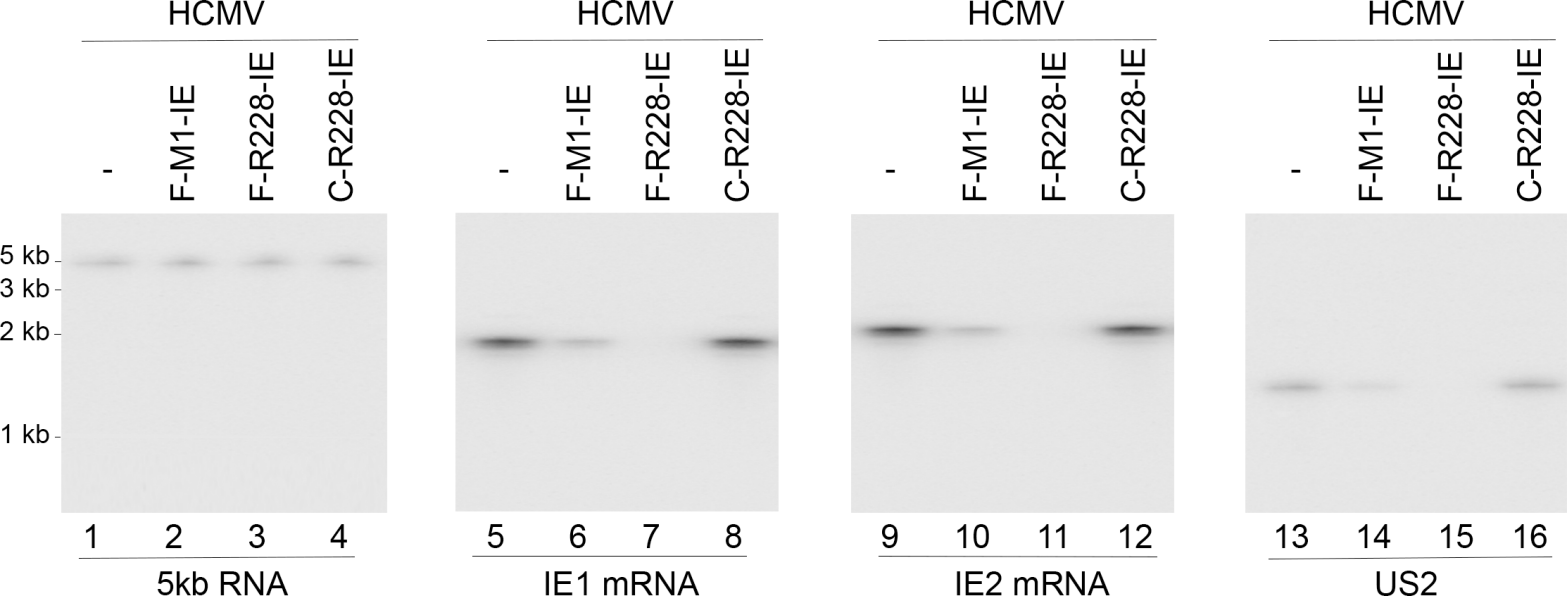
Levels of viral mRNAs assayed by Northern blot analysis. RNA fractions (45 μg) from the parental U251 cells (-) and cells expressing different ribozymes (F-M1-IE, F-R228-IE, and C-R228-IE) were hybridized to probes for detection of 5kb RNA, IE1 mRNA, IE2 mRNA, and US2 mRNA. We observed no expression of viral mRNAs in mock-infected U251 cells [30–32].

To study the ribozyme efficacy in decreasing IE1/IE2 expression, HCMV was used to infect the cells with multiplicity of infection (MOI) of 2. Northern blot experiments were used to reveal the expression levels of IE1 mRNAs (Fig 3, lanes 5–8) and IE2 mRNAs (Fig 3, lanes 9–12) in the infected cells, with the internal control being the expression levels of the 5kb long immediate-early (α) RNA (5kb RNA) (Fig 3, lanes 1–4). We observed no expression of viral mRNAs in mock-infected U251 cells [30–32] (data not shown). The expression level of this 5kb RNA transcript is not under the control by IE1 and IE2 [1]. In cells expressing F-R228-IE and F-M1-IE, IE1 and IE mRNA expressions were reduced by about 98–99% and 77–79% respectively (Table 2). In comparison, in cells expressing C-R228IE, and C-M1-IE, the IE1 and IE2 mRNA expressions were only reduced by less than 10% (Table 2). Antisense effect is probably the reason for the low inhibitory outcome found in cells expressing C-R228-IE and C-M1-IE RNAs because these control ribozymes shared the identical guide sequence as F-R228-IE and F-M1-IE. Hence, in cells expressing these ribozymes, F-R228-IE and F-M1-E may catalytically cleave the targeted mRNAs and subsequently are responsible for reducing IE1 and IE2 mRNA expression.

**Table 2:** Levels of inhibition of the mRNA and protein expression of viral genes in cells expressing ribozymes, as compared to the levels of inhibition in cells that did not express a ribozyme (U251). The values are derived from experiments that were performed in triplicate and repeated three times. The values of standard deviation for these results are less than 5%.

Viral gene	Gene class	U251	C-M1-IE	C-R228-IE	F-M1-IE	F-R228-IE	F-M1-TK
IE1 mRNA	IE	0%	6%	8%	77±7%	99±9%	0%
IE2 mRNA	IE	0%	5%	7%	79±7%	98±8%	0%
US2 mRNA	Early	0%	2%	2%	78±6%	98±9%	0%
IE1 protein	IE	0%	5%	7%	77±7%	99±8%	0%
IE2 protein	IE	0%	5%	6%	78±8%	98±9%	0%
UL99 protein	Late	0%	0%	0%	79±7%	98±8%	0%

**Fig 4:**
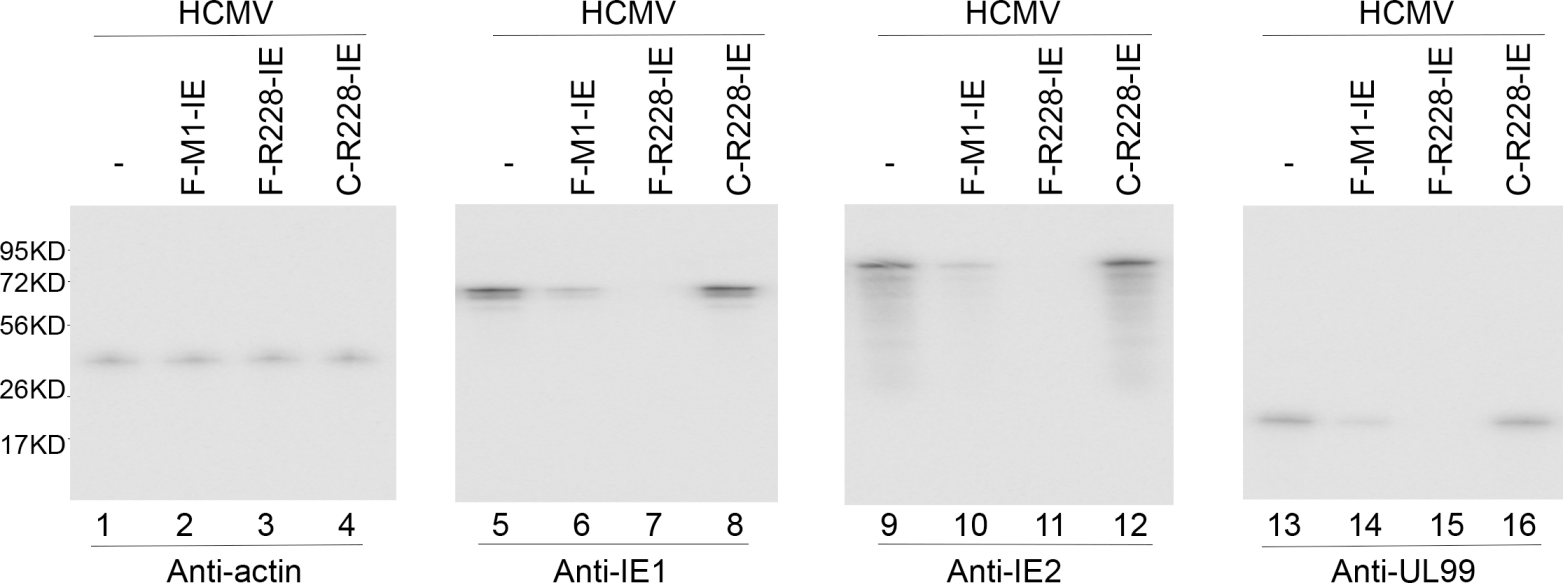
Levels of human β-actin (internal control) and viral proteins assayed by Western blot analysis. Protein fractions (60 μg) from the parental U251 cells (-) and cells expressing different ribozymes (F-M1-IE, F-R228-IE, and C-R228-IE) were reacted with antibodies for detection of human actin, IE1 protein, IE2 protein, and UL99 protein. We observed no expression of viral proteins in mock-infected U251 cells [30–32] (data not shown).

At 24–48 hours postinfection, protein samples were isolated. Western blot experiments were employed to determine HCMV IE1 and IE2 protein expression levels with the internal control being the human β-actin expression level (Fig 4, lanes 1–12). We observed no expression of viral proteins in mock-infected U251 cells [30–32] (data not shown). Table 2 summarized three independent experiments and reported that IE1 and IE2 protein expressions were reduced by 98–99% and 77–78% in cells expressing F-R228-IE and F-M1-IE RNAs. In contrast, IE1 and IE2 protein expressions were reduced by less than 10% in cells expressing C-R228-IE and C-M1-IE RNAs (Fig 4, Table 2).

### More effective inhibition of HCMV gene expression and growth in cells expressing the ribozyme variant

The expression of both viral β (early) and γ (late) genes would be reduced following repression of IE1 and IE2 proteins, which are essential for viral early and late gene expression [1]. To confirm this hypothesis, HCMV was used to infect cells with an MOI of 2 for 48–72 hours. We then measured the expression levels of US2 mRNA (a β mRNA) (Fig 3, lanes 13–16) in addition to the protein expression levels of UL99 (a γ protein) (Fig 4, lanes 13–16). The internal controls used were the levels of the HCMV 5 kb transcript and the protein levels of human actin, respectively. A drop of approximately 97–98% and 76–80% in the expression levels of IE1 and IE2 expression was documented in cells expressing F-R228-IE and F-M1-IE RNAs, respectively. Reductions in the expression of viral mRNAs and proteins were not significant in cells expressing C-228-IE and C-M1-IE (Figs 3 and 4, Table 2). The observations above imply a general repression of viral early and late genes in the cells expressing F-R228-IE and F-M1-IE.

**Fig 5:**
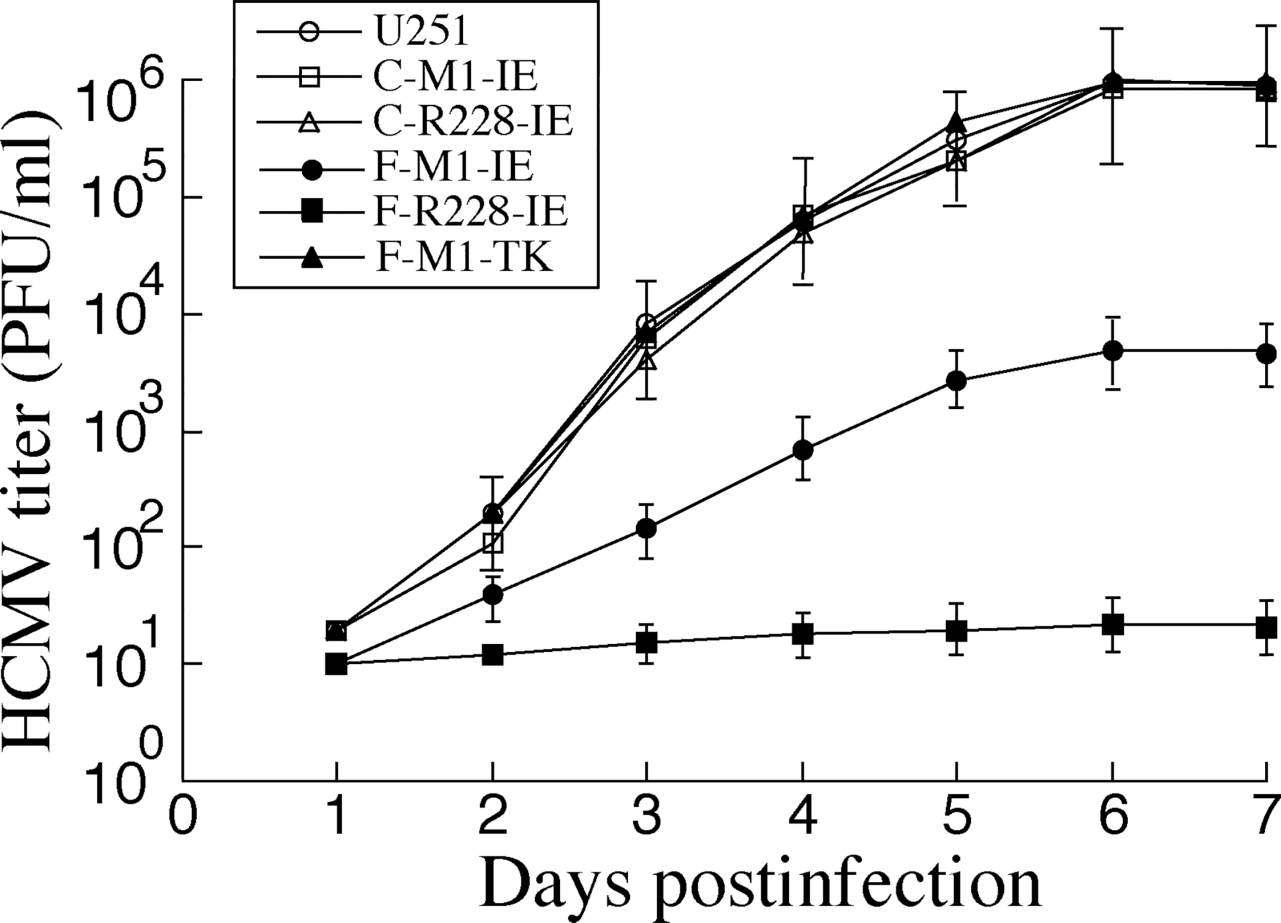
HCMV production in U251 cells and ribozyme-expressing cells. Experimental details were described in Methods. We performed the assays in triplicate and repeated the assays three times.

To establish whether viral reproduction was affected in the cells containing our engineered ribozymes, HCMV was used to infect the cells with MOI of 1. Infected cells and the cultured supernatants were collected at 24-hour intervals through 7 days post infection. Plaque forming unit was documented by determining the viral titers. Cells expressing F-R228-IE and F-M1-IE yielded 50,000 and 200-fold reduction in viral production, respectively (Fig 5). In the cells expressing the control ribozymes C-M1-IE or C-R228-IE, the reduction in viral production was insignificant (Fig 5, data not shown).

## Discussion

Ribozymes including those derived from M1 RNA have been demonstrated as potential antiviral tools capable of inhibiting gene expression and growth of viruses [3,5]. For better use of RNase P-based ribozymes as a gene-targeting tool, it is necessary to understand the working mechanism of the ribozyme and improve its cleavage activity. In this study, we demonstrated that F-R228-IE, which originated from an M1RNA variant, has higher (100 times) catalytic activity in slicing the IE1 and IE2 mRNAs *in vitro* than F-M1-IE, which originated from the wild type M1 RNA. In addition, F-R228-IE reduced HCMV IE1/IE2 expression by >98% compared with F-M1-IE, which decreased IE1 and IE2 expression by less than 80% in cultured cells. F-R228-IE decreased HCMV production by 50,000 folds while F-M1-IE reduced viral production by 200 folds. In contrast, IE1 and IE2 protein levels and viral production were only reduced by less than 10% in cells expressing C-R228-IE or C-M1-IE, which shared the same guide sequence with F-R228-IE and F-M1-IE but lacked catalytic activity (Table 1). These observations suggest that the anti-HCMV activity of F-R228-IE and F-M1-IE is largely due to the ribozyme-mediated cleavage of the targeted mRNA and not due to the antisense effect or other nonspecific effects from the guide sequence of ribozymes.

Currently, we possess little knowledge regarding the rate-limiting step of M1GS RNA to achieve ideal anti-HCMV efficacy in vivo. In our study, a region of IE1/IE2 mRNAs was targeted using our newly engineered M1GS RNAs. We hypothesized that RNase P ribozyme catalytic efficiency [(k_cat_/K_m_)^s^] will determine the effectiveness of the ribozyme cleavage and the levels of antiviral effects associated with the ribozyme in cell culture environment. If this is true, an increase in the rates of catalysis of RNase P ribozymes may result in more efficient suppression of the target mRNA expression in cultured cells. The observations in this study suggest that F-R228-IE, which is better at cleaving IE1 and IE2 mRNAs compared to F-M1-IE (originated from wild-type M1 RNA) in vitro, is also a better inhibitor of IE1/IE2 expression and HCMV production in cultured cells. Furthermore, ribozyme variant F-R228-IE, which displayed a greater value for cleavage activity [(k_cat_/K_m_)^s^], is more potent in cultured cells. These findings indicate that increasing overall cleavage rate [(k_cat_/K_m_)^s^] leads to improvement in RNase P ribozyme catalytic efficiency and subsequently enhances the ribozyme-mediated inhibitory effect of gene expression in cell culture. The difference in cleavage efficiencies of the selected variant and F-M1-IE *in vitro* (~100 fold difference) is more pronounced compared to the differences in cultured cells (98–99% vs. 77–79%). One possible explanation is that approximately 1% to 2% of targeted mRNAs might not be subject to interaction with RNase P ribozyme due to rapid transport of the mRNA to the cytoplasm.

The cleavage of the commonly shared segment of the IE1/IE2 mRNAs induced by F-R228-IE and F-M1-IE appeared to account for the antiviral effect of RNase P ribozymes. Furthermore, the enhancement in the cleavage of IE1/IE2 mRNAs by F-R228-IE may account for the higher inhibitory effects of the selected ribozyme variant on HCMV gene expression and growth. First, no significant cytotoxicity associated with the expression of ribozymes was observed as cells expressing these ribozymes had comparable growth and viability with the parental cells for up to 90 days (data not shown). Levels of human H1 RNA and actin mRNA in the ribozyme-expressing cells and parental U251 cells were similar. Second, the reduction of the IE1 and IE2 levels gave rise to the antiviral effect of the engineered ribozymes. The reason is that the expression of viral β and γ genes (US2 and UL99) was reduced in cells expressing F-R228-IE and F-M1-1E but not in those expressing C-R228-IE and C-M1-IE (Figs 3 and 4, Table 2, data not shown). We observed a correlation between the inhibition of IE1/IE2 gene expression with the subsequent observations of inhibition in the expression of viral β and γ genes. Meanwhile, we did not observe any decline in expression levels of other viral α genes (e.g. 5kb RNA) in cells expressing M1GS ribozyme (data not shown). Hence, M1GS ribozyme is specific as an inhibitor for its target mRNAs, and enhanced cleavage efficacy *in vitro* led to the enhancement of its inhibitory effects on IE1/ IE2 gene expression, overall expression of viral β and γ genes, and viral growth.

In our growth curve experiments to study the antiviral effects of the ribozymes (Fig 5), we measured the plaque forming units (PFU) of the samples prepared from the infected cultured cells. Hence, we directly assayed the number of the infectious particles (i.e. virions) produced from the cultured cells expressing the ribozymes. Another common approach to assay production of viral progeny in growth curve study is to measure the copy number of the viral DNA genome from the infected culture by quantitative PCR (qPCR). We feel that assaying the number of PFU may be more appropriate in our study since it directly assays the production of infectious virions. Additional experiments using qPCR to determine the copy number of viral DNA genome will further reveal the effects of the RNase P ribozyme variants on HCMV replication and viral production.

In this study, we showed that RNase P ribozymes can effectively block HCMV gene expression and growth in U251 cells. U251 cells are derived from neuronal tissues that are infected by HCMV in vivo [1]. These cells are permissive to HCMV replication and have been used to study HCMV infection [30–33]. It will be worth studying the effects of ribozymes in blocking HCMV infection in human foreskin fibroblasts (HFFs) that are fully permissive to HCMV infection [1]. However, HFFs are difficult to be transfected, resulting in insufficient ribozyme expression. Technically, generation of a fibroblast cell line constitutively expressing ribozymes would also be a challenge, especially after substantial cell passage, because HFFs are primary cells. On the contrary, it is easy to construct U251 cells constitutively expressing high level of ribozymes as these cells are immortalized and transfected efficiently. Further investigation on the anti-HCMV activity of RNase P ribozyme variants in other human cells including HFFs will facilitate the development of RNase P ribozymes for antiviral application.

In our previous studies [23,30–32], novel M1GS ribozyme variants, which contained unique mutations at the catalytic domain of the ribozyme, were isolated from the in vitro selection procedures and exhibited much improved activity in cleaving their targeted viral mRNAs in vitro, compared to the ribozyme derived from wildtype M1 RNA. When several of these variants were used to target HCMV essential mRNAs such as the IE1/IE2 mRNAs, we have previously observed a reduction of more than 98% in the expression of the viral targets and an inhibition of more than 10,000 fold in HCMV production in the ribozyme-expressing cells [30–32]. The results of our current study are consistent with those of previous studies, and furthermore, provide new and important insight into developing M1GS ribozymes as antiviral gene targeting agents.

Compared to those previous studies [30–32], our current study is different in the following aspects. First, F-R228-IE represented a new ribozyme variant that contained three novel mutations (G_59_ -> A_59_, C_123_ -> U_123_, and C_326_ -> U_326_) that have not been reported. In one of our previous studies, an M1GS variant, R27-IE, which contained two point mutations (U_80_ -> C_80_ and C_188_ -> U_188_), was constructed to target an overlapping region of the IE1 and IE mRNAs [32]. Second, F-R228-IE cleaved the target mRNA sequence in vitro at least 100 times more efficiently. In cultured cells, expression of F-R228-IE resulted in viral gene expression inhibition by 98%-99% and in HCMV production reduction by 50,000 folds. In one of our previous studies, RNase P variant R27-IE cleaved its target mRNA sequence in vitro about 90 times more efficiently [32]. In cultured cells, expression of R27-IE resulted in viral gene expression inhibition by 98%-99% and in HCMV production reduction by 10,000 folds [32]. F-R228-IE is among the most active and effective ribozymes found in our studies so far. Indeed, variant F-R228-IE is among the M1GS ribozymes that exhibited the highest increase of in vitro cleavage activity and led to the biggest reduction in HCMV production compared to our previous studies [30–32]. Third, the sequence of IE1 and IE2 mRNAs that was targeted by the ribozyme in our current study is different from the ribozyme-targeted sequences in previous studies. For example, in our previous study using variant R27-IE, a position 39 nucleotides upstream from the 3ꞌ terminus of exon 3 of the IE1 and IE2 mRNAs was used as the cutting site for the MG1S ribozyme [32]. In current study, we used a position 43 nucleotides downstream from the translation initiation codon of the IE1 and IE2 mRNAs as the cutting site for variant F-R228-IE. Previous studies have shown that the choice of the viral targets and the sequences with the viral target that bind to the ribozyme may significantly affect the efficacy of the ribozymes on the inhibition of viral replication and production and its efficiency in cleaving its target mRNA [16–19,30–32]. Thus, our study provides new results on the efficacy of M1GS ribozymes in targeting a unique targeting sequence in vitro and in cultured cells.

One of the most important findings in our current study is the isolation of ribozyme variant F-R228-IE. Extensive studies have been carried out to understand the catalytic mechanism and substrate binding of RNase P ribozymes [6,7]. The three-dimensional structures of M1 RNA and several other RNase P catalytic RNAs were investigated and the catalytic domain of the ribozymes includes several conserved sequences such as nucleotides 1–61 and 270–332 while the tRNA substrate binding domain includes several regions such as nucleotides 73–100 and 110–200 [9–11]. However, little is known about how M1GS ribozymes interact with a target mRNA. Compared to M1 RNA, three point mutations were found in R228: G_59_ -> A_59_, C_123_ -> U_123_, and C_326_ -> U_326_ [23]. The functions of the point mutations in the enhancement of M1GS cleavage activity are currently unknown. In one of our previous studies with variant R27, which contained two point mutations (U_80_ -> C_80_ and C_188_ -> U_188_), detailed biochemical characterization of this variant revealed that point mutation (U_80_ -> C_80_) increases the rate of chemical cleavage and point mutation (C_188_ -> U_188_) enhances substrate binding of the ribozyme [32]. Our study suggests that the three point mutations found in F-R228-IE may enhance the overall cleavage rate (k_cat_/K_m_)^s^ without altering the binding affinity (K_d_) to the mRNA substrate (Table 1). It will be interesting to determine if a ribozyme variant with these five point mutations found in both R27-IE and F-R228-IE will exhibit even better cleavage activity than R27-IE and F-R228-IE. Additional investigation of R228 and additional ribozyme variants will reveal possible mechanism(s) by which the mutations in these variants enhance the inhibitory effects of the ribozymes and offer possibilities to take advantage of these ribozymes for therapeutic applications.

## Methods

### Antibodies, viruses, and cells

The monoclonal antibody against human actin was acquired from Biodesign Inc (Kennebunk, Maine). The monoclonal antibodies against HCMV UL44, IE1, IE2, gB, and UL99 proteins were procured from Virusys (Taneytown, MD) [16,34]. Growth of human foreskin fibroblasts (HFFs) and U251 cells (American Type Culture Collection (ATCC), Manassas, VA) were supported in Dulbecco’s modified Eagle medium (DMEM) containing 10% fetal bovine serum and HCMV (Towne_BAC_) was proliferated in HFFs and U251 cells as described previously [33,35].

### Generation of substrate and ribozyme constructs

The dimethyl sulphate (DMS)-accessible regions [14,25,26] of IE1 and IE2 mRNAs were mapped following previously described procedures [16,34]. Coding sequences for functional (F-R228-IE and F-M1-IE) and control (C-R228-IE and C-MI-IE) ribozymes were derived by PCR from plasmid pFL117, pR228, and pC102, which include the M1 RNA, variant R228, and mutant C102 coding sequence respectively [23,27,36]. The 5’ and 3’ primers for the PCR reactions were Rb-IE5-AF25 (5’-GGAATTCTAATACGACTCACTATAG-3’) and Rb-IE3 (5’-CCCGCTCGAGAAAAAATGGTGACGAGGGCCCTTCCTCCTGTGGAATTGTG-3’) respectively. The coding sequence for substrate ie-39 was derived by PCR in which the 5’ and 3’ primers were ie-39-5-AF25 (5’-GGAATTCTAATACGACTCACTATAG-3’) and ie-39-3 (5’-CGGGATCCTGGAGGAAGGGCCCTCGTCAGGATTATCACCTATAGTGAGTCGTATTA-3’) respectively. In vitro transcription procedures were employed to make ribozymes and RNA substrate ie-39 and in vitro cleavage assays were performed in buffer A (50 mM Tris, pH 7.5; 100 mM NH4Cl, 100 mM MgCl_2_), following the protocols described previously [23,27,36].

### Generation of retroviral construct and ribozyme-express cells

Retroviral constructs RvF-M1-IE, RvC-M1-IE, RvC-R228-IE, and RvF-R228-IE were engineered by cloning the coding sequence for F-M1-IE, C-M1-IE, C-R228-IE, and F-R228-IE into the LXSN vector respectively, and the ribozyme expression was controlled by the U6 RNA promoter [14,27–29]. PA317 (amphotrophic cells) were transfected with M1GS-retroviral vector DNAs using a mammalian transfection (Invitrogen, Carlsbad, CA). After 2 days, supernatants from the culture, which harbored the retroviruses, were collected and subsequently used to infect U251 cells. After 2 to 3 days postinfection, cells were then selected in the presence of neomycin (600 μg/ml) (Invitrogen, Carlsbad, CA) for 14 days, and cells resistant to neomycin were propagated [14,27–29]. Northern blot analyses were performed with a probing sequence complementary to the M1 RNA sequence in order to assay the levels of M1GS RNA expression [14,27–29]. We only used cloned cells that expressed comparable levels of ribozyme in further studies.

### Viral infection and assays for viral gene expression and growth

As described previously, 5x10^5^ cells were either mock infected or infected with HCMV at a multiplicity of infection (MOI) of 2 [16,34]. We incubated the cells for the duration of time noted in the Result section. Total RNA and protein samples were collected and processed as described previously [16,34].

For Northern blot analysis to assay mRNA expression, the RNA fractions underwent electrophoresis in 1% to 2.5% agarose gels that contained formaldehyde. The RNA samples were then moved from the gel to a nitrocellulose membrane. We then used [^32^P]-radiolabeled DNA probes containing the HCMV DNA sequence for the hybridization step, and finally evaluated mRNA expression with a STORM840 phosphorimager. From plasmids pH1, pCig27, pCig38, pIE1, pIE2, and pFL117, radiolabeled DNA probes were produced to detect H1 RNA, HCMV immediate-early 5 kb RNA transcript, US2 mRNA, IE1 mRNA, IE2 mRNA, and M1GS RNAs, respectively [32,34]. To determine the protein expression levels by Western blot analysis, the denatured polypeptides from cell lysates were subjected to electrophoresis using SDS-polyacrylamide gels cross-linked with *N*,*N*''-methylenebis(acrylamide). The protein samples on the gels were then moved to the nitrocellulose membranes, and subjected to reactions with a coupled-enzyme immunoassay using anti-mouse IgG conjugated with alkaline phosphatase as well as the antibodies against HCMV IE1, IE2, UL99, and human actin. Chemiluminescence substrates from a Western chemiluminescence substrate kit (GE Healthcare, Shanghai, China and Sunnyvale, CA) were used to treat the membranes. Finally, a STORM840 phosphorimager was used to quantify the protein expression. We perform quantification in the linear range of RNA and protein detection and in triplicate experiments.

To assess the ability of the ribozymes in reducing viral replication, HCMV was used to infect the cells with MOI of 1 [32,34]. At 24-hour intervals through 7 days postinfection, the cells and supernatant were collected, and viral stocks were obtained by adding an equivalent volume of 10% non-fat dry milk and sonicating the mixture. The titers for viral samples were obtained by infecting 10^5^ HFF cells and determining the numbers of plaque-forming unit at 12 days postinfection in triplicated experiments [32,34].

### Single-turnover kinetic analyses of in vitro cleavage reactions

In buffer A (50 mM Tris, pH 7.5; 100 mM NH4Cl, 100 mM MgCl_2_), substrate ie-39 was subjected to different cleavage reactions by the ribozymes, and the values of observed cleavage rate (k_obs_) were determined by single-turnover kinetic analyses [23,32,34]. From the plot of observed cleavage rate vs. ribozyme concentration using a Kaleidagraph program (Synergy Software, Reading, PA), values of the overall cleavage rate (k_cat_/K_m_)^s^ were determined from triplicate experiments [23,32,34].

### Determination of binding dissociation constant (K_d_)

We determined the equilibrium dissociation constants (Kd) of complexes of the ribozymes and substrate ie-39 using a modified procedure from Pyle et al [37]. M1GS RNAs in different dilutions were incubated in buffer E (50 mM Tris, pH 7.5; 100 mM NH4Cl, 100 mM CaCl2, 3% glycerol, 0.1% xylene cyanol, 0.1% bromophenol blue) for 10 minutes. Then, the same volume of different dilutions of substrate ie-39 that was preheated under the same conditions was added to the ribozyme samples [23,32,34]. The binding was performed by incubating the samples for 10 minutes. Subsequently, the samples were loaded on a 5% polyacrylamide gel for electrophoresis. The running buffer for electrophoresis consisted of 100 mM Tris-Hepes pH 7.5 and 10 mM MgCl_2_ [37]. We generated a plot of percent of product bound versus M1GS RNA concentration and then extrapolated the value of K_d_ using the average values obtained from three independent experiments [23,32,34].
